# Policy change implications for forest water protection in Sweden over the last 50 years

**DOI:** 10.1007/s13280-019-01274-y

**Published:** 2019-11-08

**Authors:** Eliza Maher Hasselquist, Irina Mancheva, Katarina Eckerberg, Hjalmar Laudon

**Affiliations:** 1 Department of Forest Ecology and Management, Swedish University of Agricultural Sciences (SLU), Skogsmarksgränd, 901 83 Umeå, Sweden; 2 Department of Political Science, Umeå University, 901 87 Umeå, Sweden

**Keywords:** Forest water, Policy change, Protection zone, Riparian buffer, Riparian reserves, Water quality

## Abstract

Improving water quality has become an important environmental issue, spurred in part by the Water Framework Directive. However, the relationship of policy change with forest water protection measures is relatively unknown. We analyzed how policy and practice have developed in Sweden using 50 years of historic data from the Krycklan Catchment Study, focusing on riparian buffers. Corresponding to legislation, education and voluntary measures emphasizing stream protection, two step changes occurred; between the 1970s–1980s, buffers increased by 67%, then by 100% between 1990s and 2000s. By 2013, just 50% of the stream length affected by forestry was protected and the application has varied by stream size; small streams lacked a buffer approximately 65% of the time, while 90% of large streams had buffers. The doubling of buffer implementation from the 1990s–2000s corresponded to the adoption of a number of environmental protection policies in the 1990s that all came into effect during this period.

**Electronic supplementary material:**

The online version of this article (10.1007/s13280-019-01274-y) contains supplementary material, which is available to authorized users.

## Introduction

Protecting and improving water quality and quantity in forested landscapes has become a pertinent environmental issue to resolve in national policies globally (Gundersen et al. 2010; Richardson et al. 2012; Ring et al. 2017). Little is known, however, about the relationship between new water policies and their implementation in forest management practice. Of particular concern when trying to ensure water quality in managed landscapes is the protection of riparian zones, the terrestrial area bordering streams and rivers. Riparian zones are the interface between the aquatic and the terrestrial ecosystems that help regulate the ecological functions of both systems (Naiman and Décamps 1997). Riparian forests receive and filter water, sediment, and nutrients transported from upslope areas, thus regulating the nutrient loading to the aquatic system (Naiman and Décamps 1997; Gundersen et al. 2010). Forestry that disturbs riparian zones can affect nitrogen export, methyl mercury leakage, and erosion processes (Kreutzweiser et al. 2008; Bishop et al. 2009). Thus, riparian zones are important for ensuring the quality of water originating in our forests, even on small streams.

The smallest streams in the landscape are most likely to be impacted by forestry activities due to their abundance—representing > 75% of the total river network length (Bishop et al. 2008; Ågren et al. 2015). Furthermore, they are typically not present on available maps (Ågren et al. 2015) and often have been channelized or modified and assumed to now be drainage ditches (Hasselquist et al. 2018). Regardless of whether they are recognized as proper streams, these small, sometimes intermittently flowing, waterways are important sources of water, energy, and biological diversity that support and sustain downstream reaches (Wohl 2017), thus their disturbance likely has cumulative effects to downstream waterbodies (Kuglerová et al. 2017).

With increased demands on active forestry from different sectors for the production of bioenergy, biofuels, and climate mitigation (Söderberg and Eckerberg 2013; Lidskog et al. 2018), the volume of wood harvested from Swedish forests has increased over the last 70 years (SFA 2018). This is not because larger areas are cut (SFA 2018) but is rather due to intensified growth and extraction of wood from the same area. Riparian protection has therefore become more important than ever to ensure good water quality.

Sweden is a country dominated by forests with a reputation as an international leader in environmental policymaking and implementation (Duit 2014). This makes Sweden an interesting case to investigate forest water protection policy over time. Here, we examine if, when, and how water issues became integrated into Swedish forest and environmental policy, and what changes can be discerned in the forest landscape as a possible result thereof. We hypothesized that (1) the implementation of riparian buffer zones would increase corresponding with the implementation of each new Forestry Act and especially related to the introduction of (voluntary) forest certification in 1996. Furthermore, we hypothesized that (2) the implementation of the European Water Framework Directive (WFD) would correspond to make riparian buffer zones standard practice after the year 2000, but that (3) small streams would be less likely to receive riparian buffers than large streams due to their underrepresentation on maps.

We used a unique dataset describing historic forest management in Sweden over the last 50 years to analyze the relationship over time between the introduction of specific policy instruments for forest water protection and the effects on forest management practices, specifically riparian buffer zone protection. We complemented data on riparian buffer protection by quantifying forest clear-cuts and drainage ditching over time. The goal of collecting harvest and ditch digging data was to attain a more general picture of the intensity of forestry practices that could affect water quality and to note potential changes over time. We focused on the Krycklan Catchment Study area (KCS) in northern Sweden because it is typical of catchments dominated by Swedish forests, and thus, we could focus on the effects of forestry, while avoiding other catchment-scale stressors associated with urban areas or agriculture. Eighty-seven percent of the KCS is covered by Norway spruce and Scots pine managed for production forestry, with about 10% composed of a myriad of water bodies, and merely 3% of this area consists of farms or buildings and arable land (Laudon et al. 2013). The forests within the KCS are managed by a combination of private individuals as well as forest companies. Furthermore, the KCS has been subject to extensive research and documentation of its hydrology and water quality the last 30 years (Laudon et al. 2013). To support our results, we also used the publicly available national-scale data on area harvested and riparian buffer zones formally protected in nature conservation agreements.

## Theoretical framework

Public policymaking includes the setting of goals and developing appropriate policy instruments to meet those goals. In practice, different policy instruments are often applied together in pursuit of a set policy goal, while reinforcing each other (Bemelmans-Videc et al. 1998). As a comparison across the Nordic-Baltic region points out, legal prescriptions for protecting riparian zones vary substantially among countries with similar natural conditions (Ring et al. 2017), and legal requirements are indeed an important policy element to consider. However, real changes in forest water management might also be attributed to other more general social, technical, and economic developments than policy tools (Eckerberg 2015), pointing to the need to have a long time series of observations in order to detect patterns of interaction between policy and outcome. The growing practice of public–private partnerships as a tool for policymaking and implementation (Bjärstig and Sandström 2017), as well as voluntary forest certification (Johansson 2013) is also relevant to consider in this respect. Therefore, policy development analysis should take into account both legal and regulatory activities, economic fees, and subsidies along with the so-called ‘soft steering’ through education, information, and advice. When examining the result of public policy, we emphasize the need to apply a broad perspective on how the government might ‘steer’ societal actors. We hypothesized, in line with Bemelmans-Videc et al. (1998), that steering towards implementation of forest water protection measures indeed requires a combination of ‘hard regulatory’ instruments and ‘soft instruments,’ but that a certain time lag is likely from the introduction of a new policy instrument to detecting visible effects in forest management practice.

## Materials and methods

The study combines an analysis of Swedish policy documents and previous research on forest and water policy with an interpretation of historic aerial photos of the Krycklan Catchment Study area as well as an examination of statistical data from the Swedish Forest Agency. The study primarily covers the period from the 1960s to 2013.

### Policy analysis

For the review of policy change over time, we studied public policy development concerning forest-environmental protection, with special emphasis on protection of water environments. We analyzed government policy in the form of public investigations, bills, and legislation, as well as other types of public–private instruments such as education, information, and market-based forest certification. In particular, we focused on policymaking by the Swedish Forest Agency (SFA) and the Water Authorities (WAs).

### Krycklan Catchment Study area GIS analysis

In order to investigate whether the changes in policy had any effect on the protection of forest water, we focused primarily on one forestry practice—the protection of riparian buffers. We chose this specific practice because (1) it has a significant impact on forest water quality (2) it is relatively easy to evaluate through aerial photo interpretation. Additionally, we measured area of new clear-cuts as well as the length of forest ditches, as these practices are also closely tied to forest water quality and visible from aerial photos.

A well-trained analyst from the Swedish National Forest Inventory interpreted aerial photos; the authors then reviewed these interpretations. The Svartberget Research Park was excluded from our analysis because of the long history of forestry research and this area is likely not representative of standard forestry practice. One aerial photograph from each decade from 1963 to 2013 was interpreted to cover a 50-year time span. For each year, the boundaries of new clear-cuts were delineated in GIS (geographical information system). The smallest area classified was 0.1 hectares (ha). Forest ditches were originally digitized using a digital elevation model (DEM) created from LiDAR imagines taken in 2013 (Hasselquist et al. 2018). This first evaluation of the length of the ditch network was then compared to historical photos and the length of ditches adjusted for their presence/absence on the historical photos.

To evaluate how streams near these new clear-cuts were protected, a stream network was developed for the KCS through modeling surface flow on a DEM created from LiDAR imagines taken in 2013 (see Ågren et al. 2015 for specifics). Stream segments that where within 40 m of a new clear-cut identified for a given time period (1963–1975, 1975–1985, 1985–1993, 1993–2004, 2004–2013) were classified as either inside a protected area < 10 m; inside a protected area > 10 m wide; or without protection. For each time period, the total length of stream with each category of protection was divided by the total meters of stream affected by new clear-cuts in that year to get a proportion of stream length with a given type of riparian buffer.

The catchment area (CA) of these affected stream segments was determined by assigning values from the flow accumulation grid used to model the original stream network to each segment (Hasselquist et al. 2018). The catchment areas were then categorized into the following stream sizes, from small to large: 2–10 ha (supports flow seasonally, > 50% of the snow-free season; Ågren et al. 2015), 10–30 ha (10 ha is the average CA of streams with year-round flow, while 30 ha is the best match for streams on the most detailed Swedish maps; Ågren et al. 2015, Ågren and Lidberg 2019), 30–60 ha, 60–100 ha, 100–300 ha, 300–1000 ha, and > 1000 ha (but not more than 6700 ha because this is the CA of the KCS). For each stream size category, we summed the total length of stream affected by new clear-cuts. We then calculated the proportion of length of each of these stream size categories with a given buffer type (> 10 m, < 10 m, none).

### Comparison to the national level

Data about forest harvest and formally protected riparian buffers via either ‘habitat protection areas’ or ‘nature conservation agreement’ in Sweden (‘biotopskydd’, 7 § 11 of the Swedish Environmental Code and ‘naturvårdsavtal’ in Swedish, a civil-law agreement between a landowner and the SFA, a county administrative board or a municipality) were downloaded from the SFA’s Statistics Database (http://pxweb.skogsstyrelsen.se; December 4, 2018). To gather data on all of the formally protected riparian zones, we summed the new areas protected annually from three categories of habitat protection areas: ‘brooks and small water habitats with surrounding land,’ ‘riparian or floodplain forest,’ and ‘riparian or aquatic environments essential for threatened species.’ To this number, we also added new areas protected annually under nature conservation agreements under the category of ‘buffer zones, corridors, streams, and ravines.’ Using the area formally protected in riparian buffers divided by the area of forest clear-cut per year, we calculated the proportion of land protected compared to commercially harvested.

## Results

### Forest and water policy development

#### From production to protection

Public policy to protect and manage forests in Sweden dates back to the first Forestry Act 1903 with the introduction of compulsory regeneration after clear-cutting (Table 1) and marked the beginning of a soft regulation tradition of forestry in Sweden, which still largely prevails under the so-called “freedom with responsibility.” This implies that the law sets the minimum requirements for taking into consideration all forest values, environmental included (Appelstrand 2007). Later in 1923, legal protection of young forest stands was introduced, as a reaction to extensive exploitation by the growing forest industry. The 1948 Forestry Act regulated all forest operations in line with ‘rational forest management methods,’ however, not mentioning any environmental features. At this time, the government subsidized road network expansion to facilitate forest management and extraction of timber, as well as subsidized fertilization of unproductive wetlands to stimulate timber production. From the 1840s until the early 1990s, the government subsidized the drainage of peatlands and wetland forest via ditching, with digging of ditches peaking in the 1930s (Hasselquist et al. 2018). Ditching was seen as important not only for both frost prevention and increased wood production, but also for employment during the Great Depression (Fiskesjö and Rudqvist 2000). Digging new ditches in previously undrained forests required a permit starting in 1986 (Fiskesjö and Rudqvist 2000). However, cleaning of old ditches down to the original depth is still common practice today (SEPA 2009).

**Table 1:** Milestones in Swedish forest-environmental and water policy and their potential impacts

Year	Policy change	Main implication in relation to forest water protection	How might it affect forest water protection?
1903	First Swedish Forestry Act (Ekelund and Hamilton 2001)	To secure regeneration and professional forest management	Increased Forestry Agency (SFA) monitoring and control over forest operations
1840s–1991	Government subsidies for forest drainage but Permit for drainage introduced 1986 (Ekelund and Hamilton 2001)	Incentive to make ditches and straighten natural streams to increase timber production Disincentive to drain water-logged forest	Water-logged forest land being drained with considerable negative impact on forest water Permit for drainage likely to reduce such impact
1969	Environmental Protection Act (SFS 1969:387)	This act included general requirements for water protection, such as protection of riparian zones and avoidance of deep ruts	A general environmental policy aim to protect forest water, albeit lacking enforcement mechanisms; little impact
1979	The Forestry Act (SFS 1979:429)	Forestry Act amended to (among other things) include requirements for environmental protection, the latter originally contained in its § 21	Environmental requirements targeting forest operations, with some enforcement mechanisms, with accompanying education/information activities; some impact likely
1988	The Government’s Environment Bill establishes the principle of “sector responsibility,” legally in force from 1996 (Gov Bill 1987/88:85)	The SFA was made principally responsible for monitoring forest-environmental protection within commercial forestry	The SFA introduces a monitoring and evaluation program, making this information public and open to public debate/pressure from environmentalists
1993	“A new forest policy” (Govt Bill 1992/93:226)	The Forestry Act amended to place environment and production goals at par; environmental protection requirements in § 30	Strengthened emphasis on forest water protection by law; considerable impact likely
1995	Sweden becomes EU member	Implied that Sweden must implement EU directives relating to water protection	No immediate impact likely
1996 and onwards	Forest certification introduced in Sweden as a voluntary (market-based) policy instrument	The FSC and PEFC requiring the protection of riparian buffer zones and ‘nature consideration areas’ to become certified	Some impact likely among certified forest owners
1999	15 National Environmental Quality Objectives adopted (a 16th on climate added in 2004) (1998/99:MJU6)	‘Living Forests’ becomes the main environmental goal for the forest sector, with additional more specified intermediate targets	Generally agreed public policy goals along with their monitoring helps to increase transparency; some impact likely
1999	The Environmental Code (Ds 2000:61)	A comprehensive environmental code enters into force	Although forestry is not specifically included, some impact is likely due to its impact on increased habitat protection and nature conservation agreements
2000	The EU Water Framework Directive (WFD 2000/60/EC) enters into force in Sweden in December 2000	‘Good status’ required for waters; 5-year policy cycles with action plan The Forest Agency made responsible for promoting water protection on forest land in collaboration with Water Authorities and forest owners	Additional pressure on the forest sector to consider forest water; some impact likely in particular through education and accompanying project funding
2013	SFA prescriptions about forest water protection (SKSFS 2013:2)	Protection zones with trees and bushes should be retained in forest management to promote biodiversity and water quality etc.	Some impact likely from more concrete legal advice along with education and accompanying project funding
2014	The Forest Dialogue Process initiated by the Govt with 4 working groups consisting of public and private stakeholders	Strategic recommendations provided to the Govt on Sep 1, 2016 (dnr N2016/06464/SK). “Forest-Water Target Pictures” and recommendations for forestry to take water into account in all operations are developed	Some impact likely from increased awareness

References for Table 1 are in Appendix S1

Inspired by the growth of the environmental movement worldwide, public concern for the forest environment became a major political issue in Sweden starting in the 1960s, triggered by the introduction of large-scale modern harvest technologies. These concerns were addressed by the Swedish government with the revision of the Forestry Act in 1974 stipulating that environmental considerations must be taken within all forest operations. The SFA was given responsibility to enact more specific regulations regarding the design of clear-cuts and forest roads, the retention of trees and riparian buffer zones, fertilization and drainage, and a penalty clause was added in 1979 in case such a prescription was not followed. These changes reflected a switch in policy focus from only forest production to considering environmental protection.

These new requirements were accompanied by intensive environmental education provided both by the SFA and by non-government organizations. However, at this time, the SFA did not emphasize the need for water protection in forestry other than to avoid forest operations on mires and wetlands (SFA 1974). It was instead environmental organizations, the forest owners associations, and the Swedish Hunting Association, who stressed the need for preserving wetlands, promoting good conditions for fish and wildlife in forested ecosystems and who advocated practical measures for this purpose in their study materials (Hermansson et al. 1976). As a consequence, environmental protection became a mandatory subject in forest education at all levels as early as the 1970s, and many forest owners became engaged in study circles as a means of awareness raising about forest-environmental protection. Despite this development, research on the implementation of the new forest-environmental legislation in the following decades showed that only about half of the required environmental features were protected (Eckerberg 1990). Specifically, riparian buffer zones were the least protected within clear-cuts, with deep ruts from forest machinery commonly documented in these sensitive areas (Eckerberg 1990).

Environmental policy issues remained high on the Swedish political agenda throughout the 1980s and 1990s. The ‘sector responsibility’ was introduced in the environmental policy in 1988, whereby all sectors should be responsible for their environmental impact and work towards better environmental performance (Persson et al. 2016). Environmental protection laws were revised in the 1990s, including the enactment of the new Forestry Act in 1993. For the first time environmental and wood production goals were made equally important, requiring efficient and sustainable production while also protecting ecosystem services and other societal values. In line with previous Forestry Act, it gives the forest owner considerable leeway to decide over how those goals are implemented in practice. It is, however, a “frame law,” which means that more detailed regulations can be issued by the SFA over time (Eckerberg 1990; Appelstrand 2007). Most importantly, Sweden became a member of the European Union in 1995, with obligations to adopt and adapt Swedish environmental legislation to that of the EU (Eckerberg 2015).

The adoption of the Swedish National Environmental Quality Objectives (NEQOs) in 1999 was an important milestone in Swedish environmental policy (Persson et al. 2016). It builds upon the principle of ‘sector responsibility,’ and while the Swedish Environmental Protection Agency (SEPA) is ultimately responsible for monitoring the NEQOs, those relevant for forestry are monitored by the SFA. International agreements played an important role in pushing for environmental policy development, not least by creating political legitimacy for further action, and by requiring transparent monitoring of progress.

#### Bringing forest water to the forefront

Inspired by international agreements on public–private partnership arrangements, voluntary forest certification grew rapidly in Sweden from 1996 and onwards in pursuit of sustainable development goals. The certification schemes of the Forest Stewardship Council (FSC), and later also the Programme for the Endorsement of Forest Certification (PEFC), were easily accepted by Swedish forest owners, mostly as a way to retain market shares in the light of the strong environmental movements in Europe and elsewhere (Johansson 2013). Currently, about 2/3 of Sweden’s productive forests are certified under one of these schemes. The certification schemes require that the forest owner develops a ‘green forest management plan’ and that forest operations include conserving water resources and maintaining ecological functions, such as the protection of buffer zones along water, etc. However, similar to the forest legislation, there is no specific size designated for those buffer zones in the certification standards, but they are to be determined according to the local context (Futter et al. 2011).

In 2000, the EU Water Framework entered into force. It calls for sustainable water management to achieve the two goals of ‘good ecological status’ and ‘good chemical status,’ bringing a holistic perspective on water management. It requires monitoring the water status of all ‘water bodies’ and preventative measures, and emphasizes participation via information to all citizens, consultation with affected interests, and broad engagement by relevant public and private stakeholders in the development of management and action plans (WFD 2000). In line with the sector responsibility principle and according to the WFD, the SFA has the coordinating role for ensuring water protection within forestry. Thus, SFA took responsibility for the measures required in relation to forestry and its effect on water in the first WFD action program in 2008. In 2010, the SFA decided on its new forest water policy recommendations, which in reality were strongly based on already existing practices. It recommended the inclusion of water quality in the Forest Act, as a reason for buffer zones. Nonetheless, the SFA’s other recommendations regarding buffer zones were soft, non-legislative instruments (Keskitalo and Pettersson 2012). The application of riparian buffer zones, in particular, is still under constant debate among the different forest-environment stakeholders (Ring et al. 2017).

### The development of forest water protection in practice

#### The protection of streams using riparian buffers

**Fig. 1:**
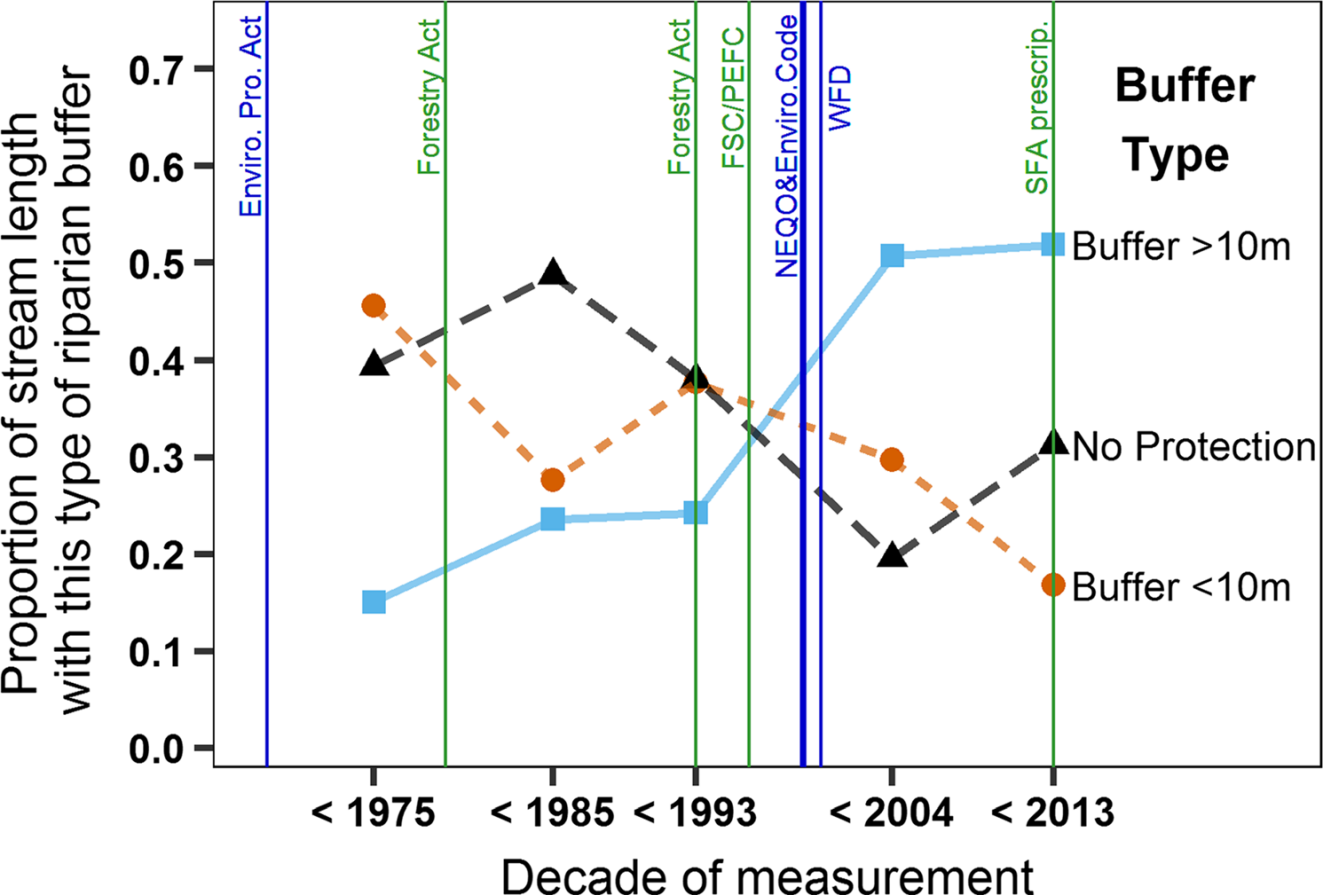
Proportion of stream length within new forest harvests that occurred between 1963–1975, 1975–1985, 1985–1993, 1993–2004, and 2004–2013 within the Krycklan Catchment Study area with either > 10 m wide protection zone (blue), no protection zone (black), or < 10 m wide protection zones (orange). Vertical lines denote timing of the milestones in Swedish forest-environmental and water policy (Table 1); green lines mark forestry-related milestones, while blue lines indicate more general environmental, or water-related milestones. In 1999, both the Swedish National Environmental Quality Objectives and the Environmental Code were adopted, hence the thicker line

The long-term perspective of the KCS dataset reveals a clear relationship between forest policy instruments and forest water protection in the form of protection of riparian buffers. These results supported our hypothesis that the application of riparian buffer zones would increase corresponding to the implementation of each new Forestry Act. Like Sweden as a whole, the area clear-cut within the KCS did not increase significantly and remained rather stable over time (Table 2), supporting KCS as representative of Sweden. The occurrence of > 10 m buffer zones was the best indicator of intentional riparian buffer retention and increased corresponding to both the 1979 and 1993 Forestry Acts (Fig. 1). Between 1965 and 1973, buffer zones > 10 m wide were left on just 15% of the length of streams affected by forest harvest (Fig. 1). After the 1979 Forestry Act, there was a corresponding 67% increase in the application of buffer zones > 10 m wide, on approximately 25% of the stream length affected by forest harvest (Fig. 1). After this, application plateaued for approximately the next two decades measured (1975–1985 and 1985–1993; Fig. 1). During the measurement period 1993–2004, the application of > 10 m buffers doubled from 25 to 50% of all streams affected by harvest in the KCS. This corresponded to the implementation of a number of considerable policy changes (Table 1), including the 1993 Forestry Act, accompanied by supportive ‘soft’ policy instruments, along with the WFD, all of which could have influenced this increase in riparian buffer application. After the flurry of policy activity in the 1990s up to 2000, our final decade of measurement (2004–2013) plateaued again in the proportion of stream length protected at about 50% (Fig. 1). Thus, our data did not support our second hypothesis, that riparian buffer zones would become standard practice corresponding to the implementation of the WFD, in 2000.

**Table 2:** Quantification of the area of new forest clear-cuts, length of new forest drainage ditches dug, and the total length of streams affected by clear-cuts in the Krycklan Catchment Study area cumulatively, and then in the five different time periods of our study

Forestry activities	Cum. Total	1963–1975	1975–1985	1985–1993	1993–2004	2004–2013
Clear-cuts (ha)	2630	407.7	851.3	230.8	711.8	428.1
Ditches dug (km)	162	4	10	0	0	0
Streams affected by clear-cuts (km)	85.8	20.8	23.9	7.5	20.0	13.5

The percentage of streams with either no protection or < 10 m buffer has generally decreased over the length of our study period, replaced by the new, wider riparian buffers. Between 1993 and 2004, > 10 m buffers finally became more prevalent than no buffer or < 10 m buffer (Fig. 1). Between 2004 and 2013, there was an increase in the proportion of streams without any buffer, from 20 to 30%; potentially signaling, the reduced influence of the WFD during this time and possibly increased pressure for a bio-based economy. The length of stream with < 10 m buffer has not changed consistently over time, likely due to these buffers being left more or less unintentionally, due to inaccessibility because of steep slopes (e.g., in a canyon), wet soils, or having some other practical barrier to harvest rather than an active choice.

**Fig. 2:**
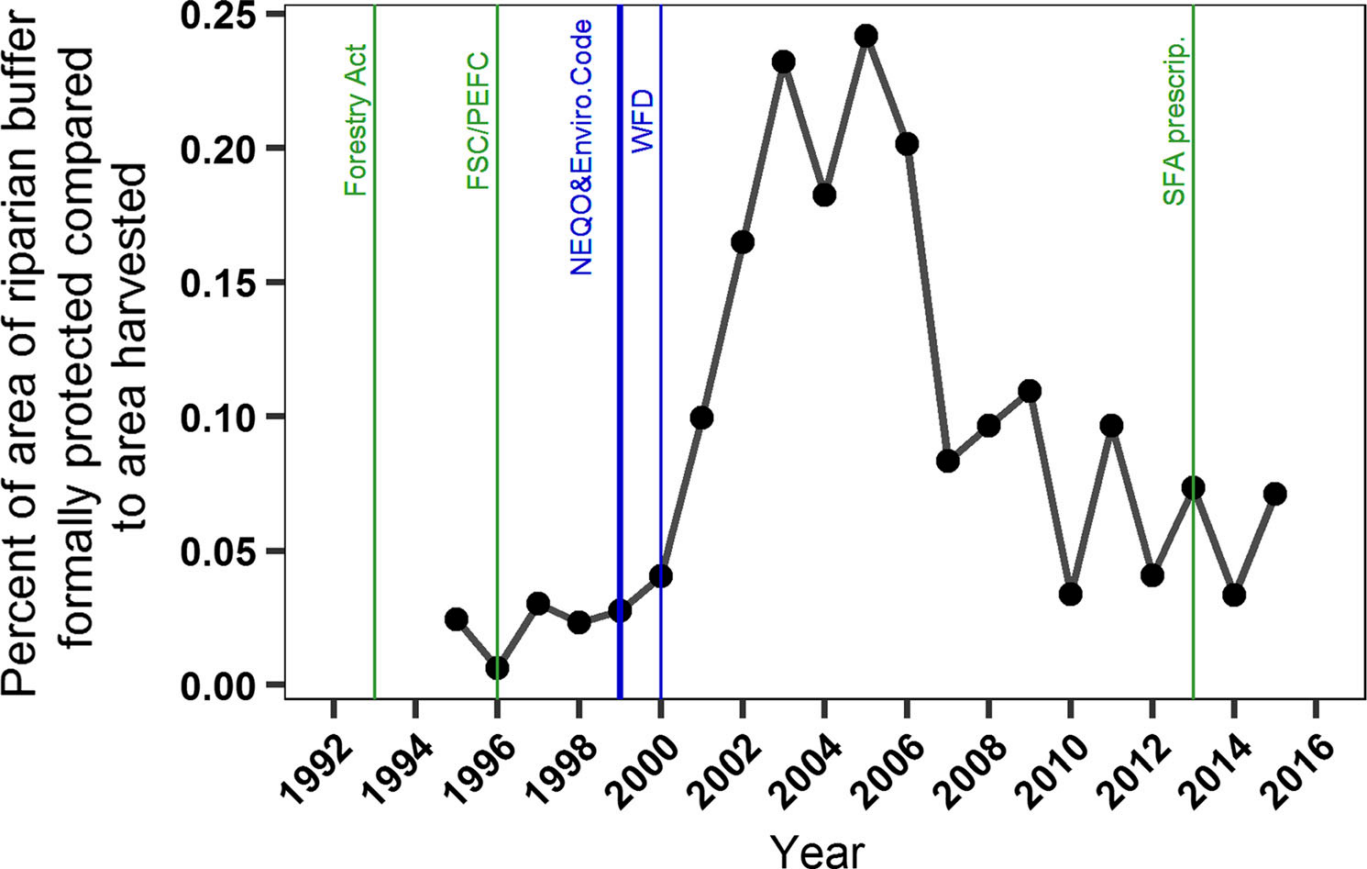
Annual percentage of new land area formally protected as riparian buffers compared to new land area of productive forest harvested in Sweden per year (formal protection means in a habitat protection area or nature conservation agreement). No publicly available data were collected by the Swedish Forest Agency about buffer zones before the 1993 Forestry Act. Vertical lines denote timing of the milestones in Swedish forest-environmental and water policy (Table 1); green lines mark forestry-related milestones, while blue lines indicate more general environmental, or water-related milestones. In 1999, both the Swedish National Environmental Quality Objectives and the Environmental Code were adopted, hence the thicker line

The detailed annual data at the national-scale revealed more about the specific importance of the 1993 Forestry act and the WFD than the decadal data from the KCS. When looking at the national-scale data (Fig. 2), we found that the SFA did not collect data about riparian buffer zones before the 1993 Forestry Act, likely signaling a change in the importance of environmental protection at the national level beginning in 1994. Using the available national-scale data, we found that the formal protection of riparian buffer zones was relatively stable from 1995 to 2000 at about 0.025% of the total area harvested (Fig. 2). Just after the flurry of policy activity in the 1990s up to 2000, there was nearly a tenfold increase in annual riparian protection, peaking in 2003 with 0.24% of the total harvested area in Sweden formally protected as riparian zones. The Environmental Code in 1999 specifically defined and created ‘habitat protection areas’ that the SFA reports on their website; many of which include riparian buffer zones. The peak protection period lasted less than 10 years (Fig. 2), and since then, formal protection of riparian buffer zones has again stabilized with an average annual percentage of 0.058% of the harvested area (Fig. 2).

#### Differential treatment of riparian buffer zones on small streams

**Fig. 3:**
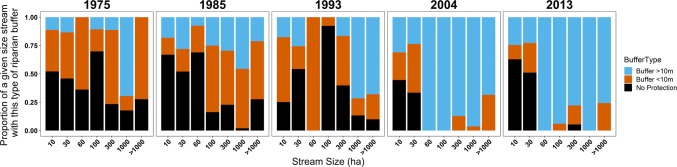
The proportion of the length of streams of different sizes within new forest harvests with different levels of protection (buffer type) within the Krycklan Catchment Study (KCS). The year above the individual plot designates the year of measurement, which includes any new forest harvests done in the previous decade (e.g., 1975 includes streams affected by new harvests done since the 1963 evaluation, 1985 includes streams affected by new harvests done since the 1975 evaluation). The stream size is based on the size of the catchment or watershed area that drains into the stream in hectares (ha). Each stream size bar is an accumulation of the sizes before them (i.e., 10 includes sizes 2–10 ha, 30 includes 10– 30 ha, 60 includes 30–60 ha, etc., but > 1000 ha can be at maximum 6700 ha—the size of the outlet of the KCS). For reference, 1 ha = 0.01 km^2^, 1000 ha = 10 km^2^

Our data supported our hypothesis that larger streams were generally better protected than small streams over the last 50 years within the KCS (Fig. 3). Interestingly, the flurry of policy activity in the 1990s up to 2000 seemed to create a strong dichotomy for which size streams were protected. Before this policy activity of the 1990s, the smaller the streams, the less likely they were to have protection, with a more or less negative linear relationship with CA (Fig. 3). Beginning in 2004, we found a shift in the pattern of protection; riparian buffers > 10 m became standard practice on large streams (> 30 ha CA) where they were implemented approximately 90% of the time. But, for small, potentially intermittent, streams (CAs < 30 ha) buffers were more the exception than the rule, with only about 25% of the stream length being protected after 2004 (Fig. 3). Given that small streams make up the majority of the stream network (Table 3), this strong dichotomy averaged out to only 50% of all streams receiving riparian buffers > 10 m (Fig. 2).

**Table 3:** Cumulative total of the length of streams of a given stream size affected by clear-cutting within the KCS over the 50-year study period. The proportion (shown as percentage) of streams affected by clear-cutting of a given stream size of a given time period are shown thereafter

Stream size (ha)	Cum. total (km)	1963–1975 (%)	1975–1985 (%)	1985–1993 (%)	1993–2004 (%)	2004–2013 (%)
2–10	30.7	28.3	41.1	25.5	45.5	29.2
10–30	18.6	16.9	24.0	13.8	25.4	23.8
30–60	4.0	5.7	4.6	4.3	3.1	5.8
60–100	4.5	7.1	4.9	12.3	2.3	3.4
100–300	12.3	19.9	8.9	27.2	10.4	14.2
300–1000	6.1	9.5	7.0	6.4	6.0	5.8
> 1000	9.6	12.7	9.5	10.5	7.4	17.9

There could be a number of reasons for this strong dichotomy in stream protection based on stream size. First, 30 ha is approximately the CA for streams found on the best available property maps (1:12 500 Lantmäteriet, Gävle; Ågren and Lidberg 2019). Ågren et al. (2015) found that 58–76% of the stream network is indeed lacking on the property map at the KCS. The primary reason for much of the stream network missing from the map is the seasonal variability of flow in the smallest streams. This could be a second reason that the smallest size class of streams (2-10 ha) are left unprotected. Ågren et al. (2015) found that streams with a 2 ha CA are flowing during 50% of the snow-free period of the year and thus could be considered ‘intermittent streams,’ but that streams with a 10 ha CA are typically flowing year-round. Hence, this smallest stream size class (2–10 ha) is likely left unprotected because of the ambiguity in the best practice recommendations. The information material developed by authorities and organizations has only recently, and very marginally, included education on small or intermittent streams. Finally, many of these smaller streams have historically been cleaned of rocks and wood and straightened and are now viewed as ditches and hence still allowed to be cleaned (SEPA 2009). Although by measuring the length of the forest drainage ditch network over time, we found that the digging of new ditches within the KCS completely stopped by 1985 (Table 2) in line with legal requirements. Our methods did not allow us to investigate whether or not existing ditches have been cleaned after that.

## Discussion

In this study, we asked how forest management policy has changed over time and whether these changes have corresponded to increased protection measures of forest water in the form of riparian buffer zones. The review of policy developments over the 50 + years showed a marked change starting around 1980. Policies that encouraged protection of forest water did not appear until the introduction of environmental protection requirements in the Forestry Act in 1979, subsidies for forest drainage ended in the late 1980s, and a permit was then required to dig new forest ditches. The ‘new forest policy’ of 1993 along with the implementation of forest certifications starting in 1996 and the adoption of the WFD in 2000 marked a further policy emphasis on forest water protection. In particular, information campaigns and education of forest owners and entrepreneurs about why and how forest water should be protected have since intensified. Importantly, most legislation in forestry policy is in the form of ‘frame laws’ that require further detailed regulation by the SFA. This means that the SFA must educate and persuade forest owners, rather than play the role of law enforcer. Similar conclusions have been made in relation to the implementation of the WFD in forestry, since this has so far generated relatively small changes in the substantial legal requirements (Keskitalo and Petterson 2012), but builds largely on voluntary measures.

In line with our hypothesis, we found clear patterns over time of the relationship between policymaking and steering through both ‘hard’ and ‘soft’ policy instruments and implementation of forest water protection in the forest landscape. Within the Krycklan Catchment, the forest sector has clearly improved the protection of streams over the course of our 50-year study period with approximately 65% of all streams affected by forestry getting some sort of riparian buffer protection by 2013 compared to about 15% in 1975. Over the 50 years, we found two distinct step changes in implementation. The first corresponded to the implementation of the 1974/1979 Forestry Act with associated changes in practice with little time lag in the 1980s, with drainage ditches no longer dug and a marked increase in protection of streams with > 10 m buffers. The second step change corresponded to the 1993 Forestry Act, but due to potential and expected time lags in implementation as well as the influence of forest certifications under the FSC and PEFC, as well as the NEQOs, the Environmental Code, and the WFD, it is unclear which of each of these policy instruments was most important. Certainly, the flurry of policy activity in the 1990s created a context that put emphasis on environmental protection and this seems to, collectively, have doubled the implementation of > 10 m riparian buffers. However, the plateauing of > 10 m buffers at 50% and the decrease in < 10 m buffers after 2000 suggests that there is still more work to be done.

The plateauing of the protection of riparian buffers may imply that those forest owners who are willing to change their practice have now done so, while those who have yet not changed may require stronger incentives and/or requirements. Nevertheless, given that we found that it is typically the small and intermittent streams that are not protected, it is likely that the policy requiring stream protection is ambiguous. Recent research has supported the importance of small and intermittent streams for downstream water quality (Wohl 2017) and the cumulative downstream effects of damages to small streams are a hot topic of research (Kuglerová et al. 2017). Many of these smaller streams have historically been straightened with rocks and wood removed and are now viewed as ditches, bringing up a broader ethical question of whether it matters that the stream was once modified if it provides habitat similar to a more natural stream. Currently, clearing of existing ditches is still permitted (SEPA 2009), which could be even more detrimental to forest water protection than not leaving a buffer (Nieminen et al. 2018).

Recently, EU and state funding has been allocated for conserving important water-related ecosystem services for the future resulting in that the forest sector in Sweden again has increased its focus on forest water quality by organizing conferences and study circles and funding the education of forest owners (Mancheva 2018). Although ‘soft steering’ educational material in recent years has included, albeit marginally, the issue of small stream protection, it is not clear from our analysis that ended in 2013 whether this will have an impact on practice. New guidelines from 2018 might improve protection of these small streams, but it may also be that this ‘soft steering’ approach through education and study circles has reached as far as it can and that ‘hard regulation’ accompanied by sanctions could be required to increase protection further.

Finally, similar to findings from Angelstam et al. (2011), the current proportion of protected riparian buffer forests in Sweden is presently too small in relation to the forest-environmental policy ambitions, which is likely to weaken their impact. Research in very large watersheds with urban and agricultural influences has shown weak water quality effects of relatively recent protection efforts like riparian buffers (Quin et al. 2015; Destouni and Jarsjö 2018), suggesting that the potential positive impacts from such forest management are not always measurable. Moreover, with predicted time lags in policy implementation, there could also be time lags in the response of water quality to mitigation due to legacy effects of historic land use, at least at large scales (Destouni and Jarsjö 2018). Thus, we have the problem of needing to protect even more forest to secure water quality (Lidskog et al. 2018), while simultaneously, climate change is placing additional demands on forests to provide biomass as a substitute for fossil fuel (Söderberg and Eckerberg 2013). Decision-makers are thus facing a complex situation, a ‘wicked problem’ (Lidskog et al. 2018), when it comes to how to handle the problem of forestry’s effects on water quality while at the same time securing other ecosystem services.

## Electronic supplementary material

Below is the link to the electronic supplementary material.

Supplementary material 1 (PDF 412 kb)
