# Supplementary material for: Policy change implications for forest water protection in Sweden over the last 50 years
Source: Ambio. 2019 Nov 8;49(7):1341–51. doi: 10.1007/s13280-019-01274-y (PMC7190599; doi:10.1007/s13280-019-01274-y)
Supplement: Supplementary file 1 — Supplementary material 1 (PDF 412 kb) [file 13280_2019_1274_MOESM1_ESM.pdf]

Electronic Supplementary Material

Appendix S1. References for Table 1 (in chronological order as seen in the Table)

Ekelund, H. and G. Hamilton. 2001. History of Forest Policy, Swedish Forestry Agency, Jönköping, http://shop.skogsstyrelsen.se/shop/9098/art45/4646045-67b381-1695.pdf (In Swedish)

SFS 1969:387. The Environmental Act, Swedish statutes, Ministry of Environment. (In Swedish)

SFS 1979:429. The Forestry Act, Swedish statutes, Ministry of Enterprise. (In Swedish)

Gov Bill 1987/88:85 on Environmental Policy for the 1990s, Government Offices. (In Swedish)

Govt Bill 1992/93:226 on a New Forest Policy, Government Offices. (In Swedish)

Government Report Environmental Policy. 1998/99:MJU6, Ministry of Environment and Agriculture. (In Swedish)

Ds 2000:61. The Swedish Environmental Code, Ministry of Environment. (In Swedish)

WFD 2000/60/EC. The European Water Framework Directive of the European Parliament and of the Council, Official Journal of the European Communities.

Swedish Forest Agency. 2013. Prescriptions SKSFS 2013:2. (Prescriptions amending Swedish Forest Agency regulations and general guidelines (SKSFS 2011: 7) regarding the Forestry Act); adopted 17 Dec 2013. (In Swedish)

Government Decision, dnr N2016/06464/SK Assignment to the Forestry Agency to assist in the implementation of the National Forest Programme, Ministry of Enterprise. (In Swedish)
